# Sequentially administrated of pemetrexed with icotinib/erlotinib in lung adenocarcinoma cell lines *in vitro*

**DOI:** 10.18632/oncotarget.23224

**Published:** 2017-12-14

**Authors:** Xiuli Feng, Yan Zhang, Tao Li, Yu Li

**Affiliations:** ^1^ Department of Respiratory Medicine, Qilu Hospital of Shandong University, Jinan, Shandong 250012, China; ^2^ Department of Respiratory Medicine, People’s Hospital of Qingzhou, Weifang, Shandong 262500, China; ^3^ The Fourth People’s Hospital of Jinan, Jinan, Shandong 250031, China

**Keywords:** lung adenocarcinoma, sequence, chemotherapy

## Abstract

Combination of chemotherapy and epidermal growth factor receptor-tyrosine kinase inhibitors (EGFR-TKIs) had been proved to be a potent anti-drug for the treatment of tumors. However, survival time was not extended for the patients with lung adenocarcinoma (AdC) compared with first-line chemotherapy. In the present study, we attempt to assess the optimal schedule of the combined administration of pemetrexed and icotinib/erlotinib in AdC cell lines. Human lung AdC cell lines with wild-type (A549), EGFR T790M (H1975) and activating EGFR mutation (HCC827) were applied *in vitro* to assess the differential efficacy of various sequential regimens on cell viability, cell apoptosis and cell cycle distribution. The results suggested that the antiproliferative effect of the sequence of pemetrexed followed by icotinib/erlotinib was more effective than that of icotinib/erlotinib followed by pemetrexed. Additionally, a reduction of G1 phase and increased S phase in sequence of pemetrexed followed by icotinib/erlotinib was also observed, promoting cell apoptosis. Thus, the sequential administration of pemetrexed followed by icotinib/erlotinib exerted a synergistic effect on HCC827 and H1975 cell lines compared with the reverse sequence. The sequential treatment of pemetrexed followed by icotinib/erlotinib has been demonstrated promising results. This treatment strategy warrants further confirmation in patients with advanced lung AdC.

## INTRODUCTION

As a life-threatening malignancy, advanced-stage non-small-cell lung cancer (NSCLC) is responsible for 17% of the total new cancer cases and 23% of the total cancer deaths, and accounts for approximately 87% of lung cancer cases [1]. Adenocarcinoma (AdC) is the most frequent subtype and rates continue to increase in most populations [2]. There exists broad evidence base supporting the administration of platinum-based combination chemotherapy has been a first-line treatment for advanced NSCLC for years [3, 4]. However, platinum-based chemotherapy leads to more gastrointestinal, neuro- or nephrotoxicity, while bone marrow toxicity is more common with carboplatin [5]. Pemetrexed, a treatment option for patients with advanced non-squamous NSCLC, had no significant differences in efficacy and safety compared with other chemotherapy options in second line treatment. It should be reasonable to optimize its use, since pemetrexed is relatively less toxic and currently used as continuation maintenance therapy for longer period, and the high budgetary impact of its incorporation into health system is of concern. Thus, optimum regimen of pemetrexed in the treatment of NSCLC should be investigated [6].

For patients with lung AdC harbouring activating epidermal growth factor receptor (EGFR) mutations, EGFR-tyrosine kinase inhibitors (EGFR-TKIs) has become the most effective first-line treatment. However, EGFR-TKI monotherapy is limited and further investigation to prolong the progression-free survival (PFS) is urgently needed. A Phase II clinical trial demonstrated that combination of pemetrexed and gefitinib was more prominent in improving PFS compared with gefitinib alone in patients with advanced nonsquamous NSCLC and activating EGFR mutations [7]. Pemetrexed was sequentially administered with gefitinib was proved to prolong a significant PFS and could be well-tolerated in advanced NSCLC patients with activation EGFR mutations [8]. In addition, a published trial conducted by Yi-Long Wu [9] confirmed that sequence of paclitaxel followed by gefitinib was an appropriate treatment regimen for AdC patients with activating EGFR mutation, indicating that the sequence of chemotherapy plus EGFR-TKIs might improve their efficacy. Although the combination of EGFR-TKIs and chemotherapy is a promising method for treating advanced AdC, the cell cycle-associated antagonistic effect is observed between EGFR-TKIs and chemotherapy [10]. It is largely due to inappropriate drug administration sequences for the negative benefits. Therefore, there is a pressing need to develop effective and viable therapeutic strategies to enhance the anti-tumor activity and prolong the survival time.

In the present study, we therefore selected the lung AdC A549 (wild-type), HCC827 (EGFR exon19 deletions), and H1975 (EGFR T790M) to investigate the treatment effects of pemetrexed, icotinib and erlotinib in different sequential schedules on cell growth proliferation, apoptosis and cell cycle distribution. Our findings may provide a meaningful insight to clinical medication.

## RESULTS

### Drug sensitivities of the A549, HCC827 and H1975 cell lines

To investigate the sensitivities of 3 drugs, A549, HCC827 and H1975 were exposed to pemetrexed, icotinib or erlotinib for 72h. Pemetrexed, icotinib or erlotinib inhibited AdC cell growth in a dose-dependent after 72 h treatment alone. Table 1 summarizes the IC50 of pemetrexed, icotinib and erlotinib. The sensitivity of A549, HCC827 and H1975 cell lines to pemetrexed was similar. The HCC827 cell line had great sensitivity to icotinib and erlotinib.

**Table 1:** IC_50_ values for each drug were calculated by performing dose-response experiments with pemetrexed, icotinib and erlotinib

Cell lines	Pemetrexed (μmol/L)	Icotinib (μmol/L)	Erlotinib (μmol/L)
A549	1.900±0.204	7.727±0.809	6.664±0.340
HCC827	1.578±0.250	0.004±0.001	0.003±0.001
H1975	3.372±0.082	5.798±0.998	4.812±0.0576

### Effects of pemetrexed, icotinib and erlotinib on cell viability

**Figure 1:**
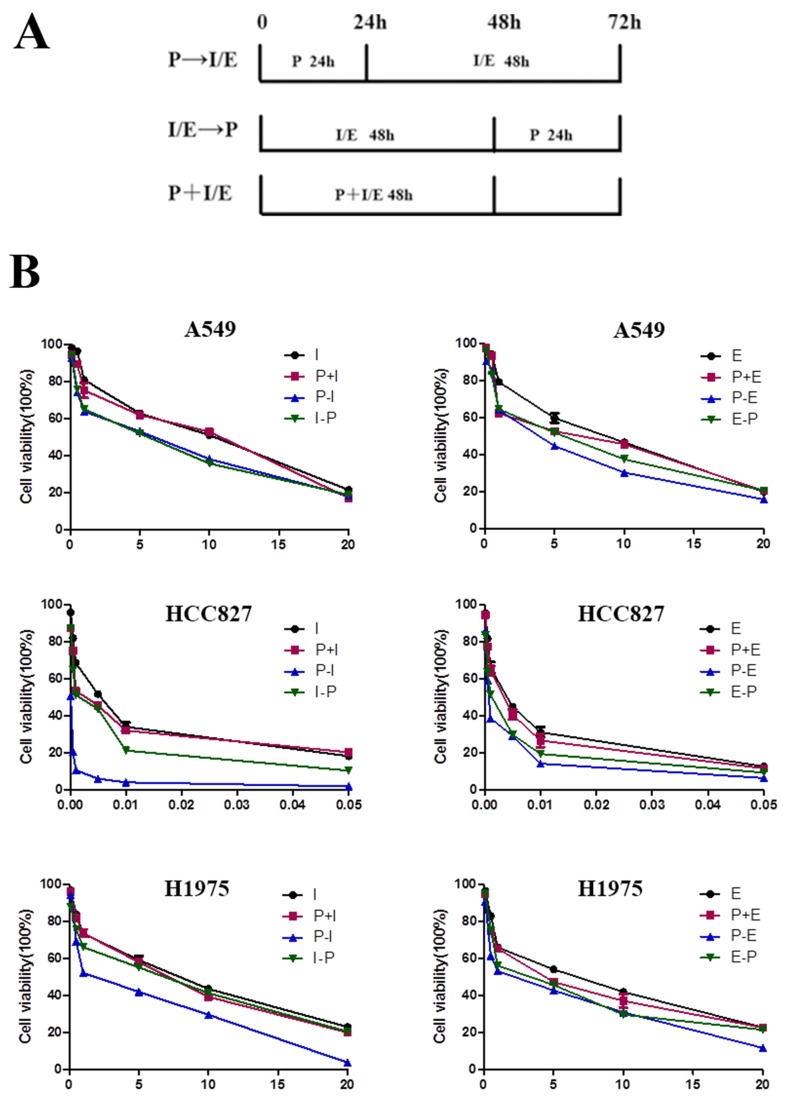
Antiproliferative effects of pemetrexed combined with icotinib/erlotinib are sequence-dependent **(A)** Schema of sequential treatment. **(B)** Sequence of pemetrexed followed by icotinib/erlotinib produced the most potent antiproliferative effect in the lung adenocarcinoma cell lines. P, pemetrexed; I, icotinib; E, erlotinib; P-I/E, pemetrexed followed by icotinib/erlotinib; I/E-P, icotinib/erlotinib followed by pemetrexed; P-I/E, pemetrexed plus icotinib/erlotinib. Data are presented as the means ± SD from three independent experiments.

Further, the cell viability of these three cell lines in three different sequences was determined using an CCK-8 assay to evaluate the antiproliferative effects of pemetrexed in combination with icotinib and erlotinib. Figure 1A showed the schema for *in vitro* sequential administration of icotinib/erlotinib and pemetrexed. As shown in Figure 1B, pemetrexed and icotinib/erlotinib have sequence-dependent antiproliferative effects. Although the differences were not significant, a superior antiproliferative effect was observed in the sequence of pemetrexed followed by icotinib/erlotinib than other sequences in the A549, HCC827 and H1975 cell lines.

The combined effects between pemetrexed and icotinib/erlotinib were determined by combination index (CI) analysis (Table 2). In the HCC827 and H1975 cell lines, the sequence of pemetrexed followed by icotinib/erlotinib exhibited a synergistic antiproliferative effect (CI<1), while CI>1 represented antagonistic effects in the sequence of icotinib/erlotinib followed by pemetrexed and the concomitant treatment (CI>1). By contrast, in A549 cell line, antagonistic activity was observed in the sequence of pemetrexed followed by icotinib/erlotinib and the reverse sequence (CI>1).

**Table 2:** Combination index (CI) values

CI	P+I	P-I	I-P	P+E	P-E	E-P
A549	1.46±0.22	1.00±0.06	1.00±0.08	1.29±0.05	1.00±0.09	1.02±0.06
HCC827	1.30±0.20	0.67±0.12	1.04±0.18	1.37±0.41	0.68±0.12	1.00±0.11
H1975	1.20±0.15	0.61±0.07	1.03±0.14	1.27±0.08	0.72±0.03	1.00±0.05

C, control group; P, pemetrexed; I, icotinib; E, erlotinib; P-I/E, pemetrexed followed by icotinib/erlotinib; I/E-P, icotinib/erlotinib followed by pemetrexed; P+I/E, pemetrexed plus icotinib/erlotinib.

### Effects of pemetrexed, icotinib and erlotinib on cell apoptosis

**Figure 2:**
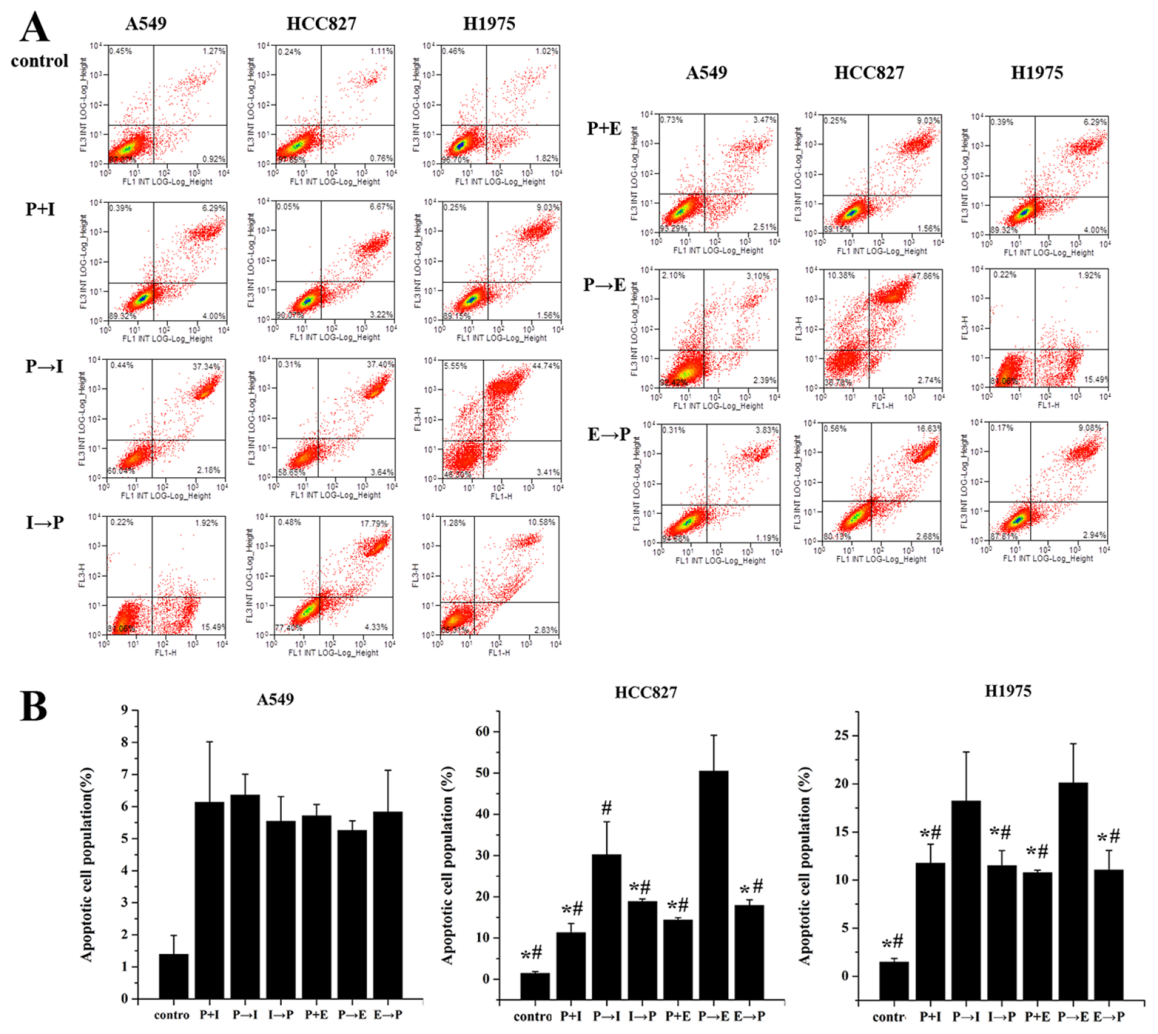
Effects of pemetrexed, icotinib and erlotinib on the cell apoptosis **(A)** Cell apoptosis was analyzed by flow cytometry. **(B)** Apoptotic and necrotic cell population were counted by flow cytometry. Cells were treated with the IC50 value of drugs. P-I/E, pemetrexed followed by icotinib/erlotinib; I/E-P, icotinib/erlotinib followed by pemetrexed; P-I/E, pemetrexed plus icotinib/erlotinib. Data are presented as the means ± SD from three independent experiments. Statistically significant differences between P-I/E and other treatment groups are presented as ^*^P<0.05.

After being exposed to pemetrexed, icotinib and erlotinib at the IC 50 values, the proportions of apoptotic cells were analyzed in Figure 2. In A549, HCC827 and H1975 cell lines, the apoptotic proportions induced by pemetrexed followed by icotinib were 6.36±0.65, 30.22±7.97 and 18.22±5.09, respectively. The apoptotic proportions in A549, HCC827 and H1975 cell lines caused by pemetrexed followed by erlotinib were 5.26±0.3, 50.47±8.68, 20.1±4.07. The apoptotic proportion in HCC827 and H1975 cell lines treated with pemetrexed followed by icotinib/erlotinib was significantly higher than other administration regimens in HCC827 and H1975 cell lines; however, there was no significant difference in A549 cell line.

### Effects of pemetrexed, icotinib and erlotinib on the cell cycle distribution

**Figure 3:**
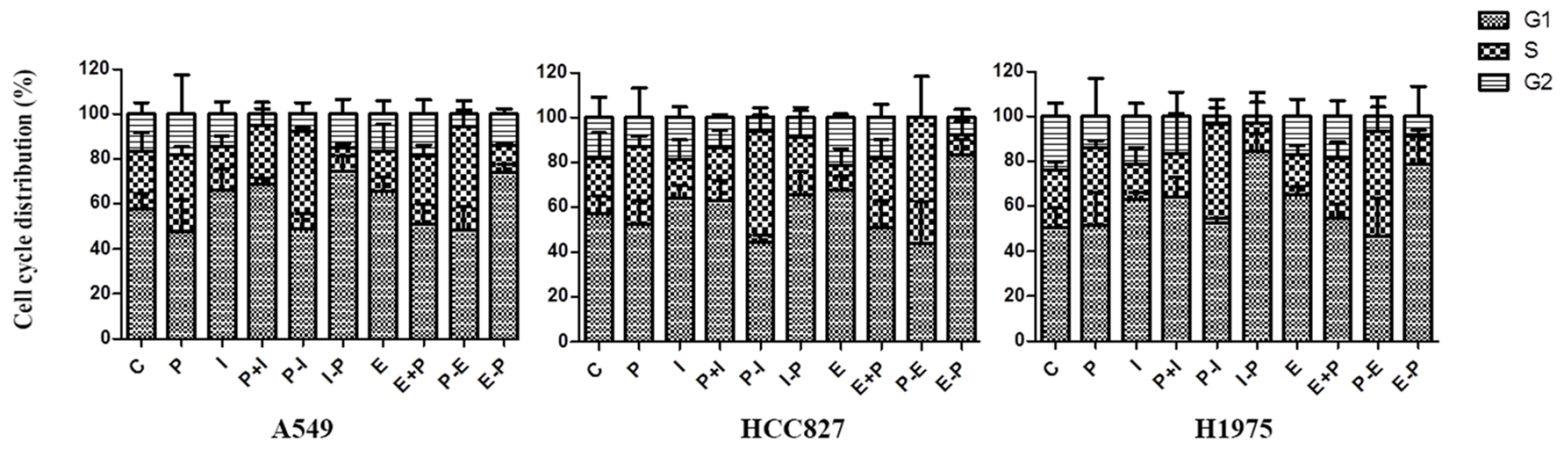
Effects of pemetrexed, icotinib and erlotinib on the cell cycle distribution Cell cycle distribution was analyzed by flow cytometry. C, control group; P, pemetrexed; I, icotinib; E, erlotinib; P-I/E, pemetrexed followed by icotinib/erlotinib; I/E-P, icotinib/erlotinib followed by pemetrexed; P-I/E, pemetrexed plus icotinib/erlotinib. Data are presented as the means ± SD from three independent experiments.

The effect on the cell cycle distribution was shown in Figure 3. In response to the sequence of pemetrexed followed by icotinib, cell fractions in the S phase were increased whereas those in the G1 or G2 phase were decreased compared with control group in A549, HCC827 and H1975 cell lines. As for the sequence of pemetrexed followed by erlotinib, cell fractions in the S phase were elevated (43.37±0.69, 49.26±2.96 and 44.05±2.93) while those in the G1 or G2 phase were reduced in A549, HCC827 and H1975 cell lines. However, decreased S phase and elevated G1 or G2 phase were found in the reverse sequence in three cell lines.

## DISCUSSION

In this present study, we conducted *in vitro* experiment to explore the optimal sequential administration of pemetrexed and icotinib/erlotinib in lung AdC A549 (EGFR wild-type), HCC827 (EGFR exon19 deletions), and H1975 (EGFR T790M) cell lines. The results showed that the antiproliferative effect of the sequence of pemetrexed followed by icotinib or erlotinib was more prominent than that of icotinib/erlotinib followed by pemetrexed. In addition, the treatment of pemetrexed followed by icotinib/erlotinib produced a synergistic effect on HCC827, H1975 cell lines. Our findings suggest that the sequential strategy is a promising approach to treat advanced lung adenocarcinoma.

EGFR-TKI combined with chemotherapy as a new strategy has become a hot research topic in the treatment of lung cancer [11, 12]. Recently, sequential regimens have attracted more interests in NSCLC researches. Fiala O et al [13] evaluated the effect of second-line pemetrexed with third-line erlotinib on advanced-stage (IIIB/IV) lung cancer with wild-type EGFR gene, indicating significant improvement of both PFS and overall survival for patients sequentially treated with erlotinib and pemetrexed compared with the pemetrexed-erlotinib sequence. A clinical retrospective study carried out by Zheng Y et al [14] demonstrated that the sequence of first-line pemetrexed followed by icotinib is promising for advanced lung cancer harboring unknown EGFR gene in China. These studies support the use of EGFR-TKIs in the second-line setting in advanced lung AdC. In the present study, sequential therapy of pemetrexed followed by icotinib/erlotinib leads to a synergistic effect on HCC827 and H1975 cell lines which is comparable to the reverse sequence of icotinib or erlotinib followed by pemetrexed.

As indicated in clinical practice, the EGFR-TKIs are recommended as first-line regimen in patients with advanced lung AdC harboring EGFR mutations. However, IPASS [15] and OPTIMAL [16] clinical trials suggested that patients with NSCLC received EGFR-TKIs alone failed to prolonged progression-free survival (PFS), and showed secondary resistance after 10-13 months in 10-13 months with objective response rate (ORR) of 43% in IPASS and 83% in OPTIMAL. In order to improve the PFS and the efficacy, JMIT study of Cheng Y found that gefitinib synchronized with pemetrexed could significantly extend PFS [7]. The FASTACT-2 study, presented by Professor Yilong Wu, showed that alternative therapy of chemotherapy and erlotinib obviously prolonged PFS and OS in patients with advanced NSCLC, especially in patients with EGFR mutations, PFS could be up to 16.8 months [17]. While, H Yan et al [18] demonstrated that concurrent chemotherapy with EGFR-TKIs was not superior to single chemotherapy or EGFR targeted therapy.

The antagonistic action between EGFR-TKI and chemotherapy seems most likely to contribute to explain these negative results. Mahaffey C M [19] performed the sequence of docetaxel followed by erlotinib in A549 and Calu-1 cell lines. The significantly enhanced apoptosis was observed compared with the reverse sequence of erlotinib followed by docetaxel, thus they hypothesized there was a negative interaction between chemotherapy and EGFR TKIs, and cell cycle arrest induced by erlotinib in the presence of wild-type EGFR accounts for the lack of benefit. An *in vitro* study performed by Cheng H et al [20] investigating differential effects of various sequences of paclitaxel with gefitinib on lung cancer cell lines with activating EGFR mutation, revealed that the sequence of paclitaxel followed by gefitinib arrested cells in G1, whereas the reverse sequence arrested cells in S and G2 phases. Moreover, it was reported that a pretreatment with EGFR-TKIs caused G1 arrest and effectively inhibited the activity of chemotherapy, resulting in decreased cytotoxicity and apoptosis [20, 21]. In the present study, single-agent icotinib/erlotinib caused accumulation of cells in G1 phase, while the reduction of G1 phase and enhanced apoptosis were observed in the sequence of pemetrexed followed by icotinib/erlotinib.

This study also has some limitations. Preliminarily, the study was only conducted *in vitro* without further confirmation, thus further evidences are needed to be provided by more *in vivo* experiments or clinical researches. In addition, more studies still need to enrich the mechanisms underlying the antagonistic interaction of these drugs.

To conclude, the present study revealed that the optimal schedule for lung AdC treatment *in vitro* was the sequence of pemetrexed followed by icotinib/erlotinib. These results may provide a rationale and promising strategy for the ongoing clinical investigation of the sequential treatment in patients with lung AdC.

## MATERIALS AND METHODS

### Drugs

Icotinib standard substance (Beida Pharmaceutical co., Zhejiang, China) was configured as 10mmol/L solution in dimethyl sulfoxide (DMSO; Sigma, St. Louis, MO, USA). Erlotinib standard substance (Roche pharmaceutical Ltd., Basel, Switzerland) was diluted with DMSO as stocking solution of 100mmol/L. Pemetrexed (Qilu pharmaceutical company, Jinan, Shandong, China) was dissolved in physiological saline and configured as 10 mmol/L stocking solution. The solutions were stored at -20°C.

### Cell lines

A549, HCC827 and H1975 human lung AdC cell lines were provided by the Chinese academy of medical sciences tumor cells bank (Beijing, China). The cell lines were cultured in RPMI-1640 and DMEM mediums (Gibco, Uxbridge, UK) containing 10% fetal bovine serum (Gibco, Uxbridge, UK) and 100 U/ml gentamicin (GIBCO Laboratories Inc., NY, USA) at 37 °C in a humidified condition containing 5% CO_2_. Cells were harvested during the exponential growth phase by trypsin.

### Evaluation of cell viability by CCK-8

The CCK-8 assay (Best Bio, Shanghai, China) was applied to evaluate the cell viability. Cells were implanted 8,000/well in 96-well plates for 24h. To evaluate the effects of single medication, the cells were exposed to icotinib, erlotinib or pemetrexed alone for 72h. To determine the antiproliferative efficacy of the combined treatment, three different sequences were performed as following: 1) Pretreated with icotinib/erlotinib for 48h subsequently treated with pemetrexed for 24h; 2) Preprocessed with pemetrexed for 24h followed by icotinib/erlotinib for 48h; 3) Treated icotinib/erlotinib simultaneously with pemetrexed for 48h, and incubated in drug-free medium for 24h. After 72h drug administration, the cells were incubated with 25 μl CCK-8 (5 mg/ml) after PBS washing at 37°C for 4h. Cell viability was detected at 570 nm. Measurements were carried out on at least 3 different spots. The half maximal inhibitory concentration (IC_50_) was determined at a concentration of 50% cell growth inhibition compared with the untreated control cells.

### Evaluation of apoptosis

Cells were planted in 6-well plates with a density of 2x10^5^/well. Then cells were trypsinized to analyze the cell apoptosis after the drug administration. 400 μl AnnexinV suspension cells were added into the cells and washed with cold PBS. Annexin V-fluorescein isothiocyanate (FITC) and 5 μl PI (Joincare Medicine Company, Zhuhai, Guangdong, China) were used for staining for 15 min at 37°C in the dark and analyzed using a FACSCalibur flow cytometer (Becton Dickinson and company, San Jose, CA).

### Analysis of cell cycle

Cells were treated with icotinib, erlotinib and pemetrexed sequentially after culturing with 0.1 FBS for 24h. Cells were trypsinized, fixed with 70% ice-cold ethanol for 2h, then were harvested by centrifugation, washed once with cold PBS, stained with 20 μl Rnase at 37 °C and 400 μl propidium iodide (PI) at 4 °C for 30 min in dark. Analysis of cell cycle was performed by flow cytometer.

### Statistical analysis

SPSS 19.0 software (SPSS, Chicago, USA) was used to all statistical analysis. Data are expressed as the means ±SD. The statistical analyses between groups were performed using one-way ANOVA test. The significance level was set at α = 0.05.

## Abbreviations

EGFR-TKIs: epidermal growth factor receptor-tyrosine kinase inhibitors
NSCLC: non-small cell lung cancer
CI: Combination index
IC50: half maximal inhibitory concentration
P: pemetrexed
I: icotinib
E: erlotinib
P-I/E: pemetrexed followed by icotinib/erlotinib
I/E-P: icotinib/erlotinib followed by pemetrexed
P+I/E: pemetrexed plus icotinib/erlotinib

## References

[1] Jemal A, Bray F, Center MM, Ferlay J, Ward E, Forman D (2011). Global cancer statistics. CA Cancer J Clin.

[2] Lortet-Tieulent J, Soerjomataram I, Ferlay J, Rutherford M, Weiderpass E, Bray F (2014). International trends in lung cancer incidence by histological subtype: adenocarcinoma stabilizing in men but still increasing in women. Lung Cancer.

[3] Rizvi NA, Hellmann MD, Brahmer JR, Juergens RA, Borghaei H, Gettinger S, Chow LQ, Gerber DE, Laurie SA, Goldman JW (2016). Nivolumab in combination with platinum-based doublet chemotherapy for first-line treatment of advanced non–small-cell lung cancer. J Clin Oncol.

[4] Soria JC, Mauguen A, Reck M, Sandler A, Saijo N, Johnson D, Burcoveanu D, Fukuoka M, Besse B, Pignon JP (2012). Systematic review and meta-analysis of randomised, phase II/III trials adding bevacizumab to platinum-based chemotherapy as first-line treatment in patients with advanced non-small-cell lung cancer. Ann Oncol.

[5] Novello S, Barlesi F, Califano R, Cufer T, Ekman S, Levra MG, Kerr K, Popat S, Reck M, Senan S (2016). Metastatic non-small-cell lung cancer: ESMO Clinical Practice Guidelines for diagnosis, treatment and follow-up. Ann Oncol.

[6] Pérez-Moreno MA, Galván-Banqueri M, Flores-Moreno S, Villalba-Moreno Á, Cotrina-Luque J, Bautista-Paloma FJ (2014). Systematic review of efficacy and safety of pemetrexed in non-small-cell-lung cancer. Int J Clin Pharm.

[7] Cheng Y, Murakami H, Yang PC, He J, Nakagawa K, Kang JH, Kim JH, Wang X, Enatsu S, Puri T (2016). Randomized phase II trial of gefitinib with and without pemetrexed as first-line therapy in patients with advanced nonsquamous non–small-cell lung cancer with activating epidermal growth factor receptor mutations. J Clin Oncol.

[8] Yoshimura N, Kudoh S, Mitsuoka S, Yoshimoto N, Oka T, Nakai T, Suzumira T, Matusura K, Tochino Y, Asai K (2015). Phase II study of a combination regimen of gefitinib and pemetrexed as first-line treatment in patients with advanced non-small cell lung cancer harboring a sensitive EGFR mutation. Lung Cancer.

[9] Cheng H, An SJ, Dong S, Zhang YF, Zhang XC, Chen ZH, Wu YL (2011). Molecular mechanism of the schedule-dependent synergistic interaction in EGFR-mutant non-small cell lung cancer cell lines treated with paclitaxel and gefitinib. J Hematol Oncol.

[10] Mok TS, Wu YL, Yu CJ, Zhou C, Chen YM, Zhang L, Ignacio J, Liao M, Srimuninnimit V, Boyer MJ (2009). Randomized, placebo-controlled, phase II study of sequential erlotinib and chemotherapy as first-line treatment for advanced non–small-cell lung cancer. J Clin Oncol.

[11] Laurila N, Koivunen JP (2015). EGFR inhibitor and chemotherapy combinations for acquired TKI resistance in EGFR-mutant NSCLC models. Med Oncol.

[12] Goss GD, Spaans JN (2016). Epidermal growth factor receptor inhibition in the management of squamous cell carcinoma of the lung. Oncologist.

[13] Fiala O, Pesek M, Finek J, Benesova L, Bortlicek Z, Minarik M (2013). Sequential treatment of advanced-stage lung adenocarcinoma harboring wild-type EGFR gene: second-line pemetrexed followed by third-line Erlotinib versus the reverse sequence. Anticancer Res.

[14] Zheng Y, Fang W, Deng J, Zhao P, Xu N, Zhou J (2014). Sequential treatment of icotinib after first-line pemetrexed in advanced lung adenocarcinoma with unknown EGFR gene status. J Thorac Dis.

[15] Fukuoka M, Wu YL, Thongprasert S, Sunpaweravong P, Leong SS, Sriuranpong V, Chao TY, Nakagawa K, Chu DT, Saijo N (2011). Biomarker analyses and final overall survival results from a phase III, randomized, open-label, first-line study of gefitinib versus carboplatin/paclitaxel in clinically selected patients with advanced non–small-cell lung cancer in Asia (IPASS). J Clin Oncol.

[16] Zhou C, Wu YL, Chen G, Feng J, Liu XQ, Wang C, Zhang S, Wang J, Zhou S, Ren S (2011). Erlotinib versus chemotherapy as first-line treatment for patients with advanced EGFR mutation-positive non-small-cell lung cancer (OPTIMAL, CTONG-0802): a multicentre, open-label, randomised, phase 3 study. Lancet Oncol.

[17] Wu YL, Lee JS, Thongprasert S, Yu CJ, Zhang L, Ladrera G, Srimuninnimit V, Sriuranpong V, Sandoval-Tan J, Zhu Y (2013). Intercalated combination of chemotherapy and erlotinib for patients with advanced stage non-small-cell lung cancer (FASTACT-2): a randomised, double-blind trial. Lancet Oncol.

[18] Yan H, Li H, Li Q, Zhao P, Wang W, Cao B (2015). The efficacy of synchronous combination of chemotherapy and EGFR TKIs for the first-line treatment of NSCLC: a systematic analysis. PLoS One.

[19] Mahaffey CM, Davies AM, Lara JPN, Pryde B, Holland W, Mack PC, Gumerlock PH, Gandara DR (2007). Schedule-dependent apoptosis in K-ras mutant non–small-cell lung cancer cell lines treated with docetaxel and erlotinib: rationale for pharmacodynamic separation. Clin Lung Cancer.

[20] Cheng H, An SJ, Zhang XC, Dong S, Zhang YF, Chen ZH, Chen HJ, Guo AL, Lin QX, Wu YL (2011). *In vitro* sequence-dependent synergism between paclitaxel and gefitinib in human lung cancer cell lines. Cancer Chemother Pharmacol.

[21] Wu M, Yuan Y, Pan YY, Zhang Y (2014). Antitumor activity of combination treatment with gefitinib and docetaxel in EGFR-TKI-sensitive, primary resistant and acquired resistant human non-small cell lung cancer cells. Mol Med Rep.

